# The association of Treg and Th17 cells development factors and anti-TPO autoantibodies in patients with recurrent pregnancy loss

**DOI:** 10.1186/s13104-023-06579-6

**Published:** 2023-10-31

**Authors:** Mitra Niafar, Vajihe Samaie, Mohammad Sadegh Soltani-Zangbar, Roza Motavalli, Sanam Dolati, Shahla Danaii, Amir Mehdizadeh, Mehdi Yousefi

**Affiliations:** 1 Endocrine Research Center, Tabriz University of Medical Sciences, Tabriz, Iran; 2 Student Research Committee, Tabriz University of Medical Sciences, Tabriz, Iran; 3 Department of Immunology, Faculty of medicine, Tabriz University of Medical Sciences, Tabriz, Iran; 4 Stem Cell Research Center, Tabriz University of Medical Sciences, Tabriz, Iran; 5 Department of Molecular Medicine, Faculty of Advanced Medical sciences, Tabriz University of Medical Sciences, Tabriz, Iran; 6 Physical Medicine and Rehabilitation Research Center, Aging Research Institute, Tabriz University of Medical Sciences, Tabriz, Iran; 7 Gynecology Departments, ACECR ART Center, Eastern Azerbaijan Branch of ACECR, Eastern, Tabriz, Iran; 8 Hematology and Oncology Research Center, Tabriz University of Medical Sciences, Tabriz, Iran

**Keywords:** Thyroid autoimmunity, Interleukin-17, TGFβ, Pregnancy loss, Th17, Tregs

## Abstract

**Objectives:**

Thyroid autoimmunity is considered as the most prevalent autoimmune condition in women in fertility age. There are different clinical evidences indicating the association between thyroid autoimmunity and increased risk of RPL. This study aimed to analyze the association of Tregs and Th17 cells development factors and anti–thyroid peroxidase (anti-TPO) antibodies in RPL patients. Healthy controls (n = 36), TPO + controls (n = 25) and TPO + RPL (n = 32) participated in this study. After blood sampling, the frequency of Th17 and Tregs was evaluated using flow cytometry. Real-time PCR and ELISA was used to assess the status of Tregs and Th17 related transcription factors and cytokines in mRNA and protein level, respectively.

**Results:**

TPO + RPL group showed a higher Th17 frequency compared to healthy controls and TPO + controls groups (p = 0.0002 and p = 0.04, respectively). Additionally, mRNA expression levels of RORγT and IL-17 were significantly higher in TPO + RPL compared to healthy controls and TPO + controls groups. In contrast, Foxp3 and TGFβ expression was lower in TPO + RPL. ELISA findings also indicated a significantly higher IL-17 and lower TGFβ secretion in TPO + RPL compared to healthy controls and TPO + controls. Thyroid autoimmunity should intensely be controlled specially in patients with RPL history.

**Supplementary Information:**

The online version contains supplementary material available at 10.1186/s13104-023-06579-6.

## Introduction

Recurrent pregnancy loss (RPL) is characterized by at least two and/or more sequential loss of pregnancy and recent evidences have indicated the maternal immune system interference in 95% of RPL patients with normal embryo karyotype [1]. It has been reported that 50% of the primary abortions are due to chromosomal abnormalities. Other causes include anatomical abnormalities (12–16%), maternal infections (0.5-5%), endocrine disorders (17–20%) such as diabetes, hypothyroidism and luteal phase failure, autoimmune disorders (20–50%) such as anti-phospholipid antibody, and environmental factors including smoking, alcohol consumption, radiation, trauma, cardiovascular, and hematological diseases [2–5].

Antibodies against thyroid peroxidase (TPO) and thyroglobulin (TG) known as anti-microsomal antibodies have been detected in Hashimoto’s thyroiditis, Graves’ disease [6]. Recent findings have also indicated that thyroid autoimmune disorders are associated with increased RPL rate [7, 8].

Recent evidences indicate that T helper 17 (Th17) and regulatory T (Tregs) imbalance play a pivotal role in idiopathic abortions [9–11]. Tregs secrete interleukin-10 (IL-10), transforming growth factor beta (TGF-β) and IL-35 as anti-inflammatory cytokines. The exposure of uterus to seminal fluid induces the CD4 + T cells differentiation into Tregs, leading to uterus environment to be rife with these cells. Tregs, then migrate to the endometrium to mediate a successful implantation [12]. Increased Th17 cytokines (IL-21, IL-17 and IL-23) level in these patients has led to neutrophil rich inflammation in uterus and consequent embryo growth inhibition and abortion [13]. The serum and decidual IL-17, IL-23 and retinoid orphan receptor C (RORC) levels were also significantly higher in RPL patients. Additionally in ex vivo culture, Tregs of RPL patients showed lower suppressive activity against IL-17 production compared to controls [14].

A group of studies have also indicated a correlation between thyroid autoimmunity and increased Th17 and decreased Tregs cells frequency [15–17]. However, there are limited information regarding the association between immunologic factors and anti-thyroid antibodies and abortion. Therefore, this study aimed to evaluate the association of Tregs and Th17 cells frequency, development factors and anti-TPO antibodies in RPL patients.

## Main text

## Materials and methods

### Study design

In this cross-sectional descriptive study, healthy controls (healthy women with successful pregnancy history, n = 36), TPO + controls (anti-TPO positive women with successful pregnancy history, n = 25) and TPO + RPL (anti-TPO positive women with primary RPL history, n = 32) were enrolled. The inclusion criteria were willingness to cooperate, age between 18 and 40 years, history of at least two miscarriages and no immunotherapy background. Age below 18 and above 40 years, abnormal karyotype in women or their husbands, coagulation disorders, chronic diseases, history of asthma and allergy, intrauterine anomalies and unwillingness to cooperate were also considered as exclusion criteria. None of individuals received levothyroxine in this study.

### PBMCs isolation

For the isolation of peripheral blood mononuclear cells (PBMCs), 10 ml of blood were obtained from individuals under sterile condition and heparinized for PBMCs isolation by Ficoll (lymphosep) 1.077 g/ml (Biowest, France) with the gradient centrifugation (450 g, 25 min) technique. Then, Isolated PBMCs were cultured in 5 ml RPMI 1640 medium supplemented with L-glutamine (200 mM), fetal calf serum (FCS 10%), 10 ng/ml of phorbol myristate acetate (PMA) (STEMCELL Technologies, Cambridge, UK), and 100 U/ml penicillin for Tregs and Th17 cells cultivation. Cells were then incubated for 48 h at 37°^C^ and 5% CO_2_. The supernatant of cell culture was used for the measurement of IL-17 and TGFβ using enzyme-linked immunosorbent assay (ELISA); while, real-time polymerase chain reaction (RT-PCR) was used for Th-17 and Tregs related factors expression.

### Flow cytometry analysis

In this study, flow cytometry was used to evaluate the frequency of Tregs and Th17 by identifying the percentage of CD25 + CD4 + CD127- and CD4 + IL-17 + cells, respectively. For Th17 cell detection, PBMCs were incubated with PMA (10 ng/ml) and ionomycin (0.5 µM, Sigma-Aldrich, Germany) for 5 h at 5% CO2 and 37 °C in the incubator. After washing, cells were incubated with fluorescein isothiocyanate (FITC)-conjugated anti-CD4 antibody (Catalog No: 566,911) for 15 min at 4 °C. FITC Mouse immunoglobulin G1 (IgG1) Kappa (Catalog No: 555,748) was also used as isotype control. Then, cells were washed twice by permeabilization/fixation buffer. Anti-IL-17-PE antibody (Catalog No: 560,436) was used for intracellular staining of the cells following by 20 min incubation at room temperature. PE Mouse IgG1 Kappa Isotype control (Catalog No: 551,436) was used as isotype control.

In order to assess the Tregs frequency, anti-CD4-FITC (Catalog No: 566,911), anti-CD25-phycoerythrin (PE) (Catalog No: 341,009), and anti-CD127- adenomatous polyposis coli protein antibody (APC) (Catalog No: 565,185) antibodies were used for 1 × 10^6^ PBMCs staining at 4 °C for 45 min. After incubation, cells were washed and resuspended in fluorescence-Activated Cell Sorting (FACS) solution, and analyzed at the same day via flow cytometer instrument (BD FACSCalibure, San Jose, USA). FITC Mouse IgG1 Kappa (Catalog No: 555,748), PE Mouse IgG1 Kappa (Catalog No: 551,436) and APC-R700 Mouse IgG1 Kappa (Catalog No: 564,974) were used as isotype controls for anti-CD4-FITC, anti-CD25-PE and anti-CD127-APC antibodies, respectively.

### Real-time PCR

RNX-PLUS Solution (Sina Clon, Iran) was employed for total RNA extraction from T cells. Complementary DNA (cDNA) was synthesized with random hexamer primers and oligo (dT) by using M-MLV Reverse Transcriptase kit (Sigma Aldrich, USA). Real-time PCR was carried out to measure the expression level TGF-β, Foxp3, IL-17, and RORγT in Tregs and Th17 cells. In this technique, gene-specific primers (OD = 2, 4µM, Sina Clon, Iran) and SYBR Green were used in the Corbett research RG-6000 real-time rotary analyzer PCR machine (Corbett Research, Bosch Institute).

### ELISA

The level of serum TSH (Pishtazteb, Iran, Catalog No.: PT-TSH-96, Reference Interval: 0.32–5.2 mIU/L), anti-TPO (Pishtazteb, Iran, Catalog No.: PT-Anti TPO IgG-96, Reference interval: up to 39.3 IU/ml), secreted IL-17 (MyBioSource, CA, Catalog No: MBS764076), and TGF-β (MyBioSource, CA, Catalog No: MBS266143) by PBMCs in media supernatant was assessed based on the manufacturer’s protocol by ELISA kits. The optical densities were measured at 450 nm by a BP-800 micro plate ELISA reader system (Biohit, Finland).

### Statistical analysis

Statistical analysis was done using Statistical Package for the Social Sciences (SPSS) (version 24.0; SPSS Inc.). Shapiro-Wilk test was used as normality test in order to assess the data distribution. Analysis was done using ANOVA flowed by Turkey’s post-hoc test between three groups. For qualitative data analysis, Chi-square or fisher’s exact tests were used. All graphs were illustrated by GraphPad Prism version 8.00 (GraphPad, CA, USA). P < 0.05 was considered as statistically significant.

## Results

### General characteristics of the population under study

Table 1 shows the general characteristics of the individuals. Thyroid stimulating hormone (TSH) level was significantly higher in TPO + controls and TPO + RPL compared to healthy controls (4.83 ± 1.82 mIU/L and 5.41 ± 1.38 mIU/L versus 2.12 ± 1.02 mIU/L, p = 0.0019 and p < 0.0001, respectively).

**Table 1:** General characteristics of the individuals

Parameter		Healthy controls (n = 36) mean ± SD	TPO + controls (n = 25) mean ± SD	TPO + RPL (n = 32) mean ± SD	*P* value		
					TPO + controls vs. TPO + RPL	Healthy controls vs. TPO + RPL	Healthy controls vs. TPO + controls
Age		29.86 ± 5.12	33.22 ± 4.64	32.81 ± 5.01	NS	NS	NS
Weight (kg)		64.96 ± 12.1	69.71 ± 11.9	68.32 ± 9.81	NS	NS	NS
Height (cm)		157.3 ± 5.61	158.8 ± 6.66	160.3 ± 5.92	NS	NS	NS
BMI (kg/m^2^)		26.25 ± 3.86	27.4 ± 3.98	26.58 ± 3.62	NS	NS	NS
Number of miscarriages			0.28 ± 0.6137	3.226 ± 0.8046	< 0.0001		
Number of live births		2.000 ± 0.8619	1.480 ± 0.6532				0.0096
RPL type	Primary			19			
	Secondary			13			
TSH (mIU/L)		2.12 ± 1.02	4.83 ± 1.82	5.41 ± 1.38	NS	< 0.0001	0.0019
Anti-TPO		0	25	32	NS	< 0.0001	< 0.0001
Smoking		0	0	0			
Hormonal Diseases		8	16	19	NS	0.002	0.001
Diabetes		3	3	6	NS	NS	NS
Liver Diseases		0	2	4	NS	0.029	NS
Thyroid Diseases		0	25	32	NS	< 0.0001	< 0.0001
Genetic Diseases		0	1	0	NS	NS	NS
Chronic Infection		0	0	0			
Chronic Inflammation		0	0	0			
Heart Problem		0	0	0			
Kidney Problem		0	0	0			
Anxiety		10	7	11	NS	NS	NS

Data are presented as mean ± standard division (SD). p < 0.05 was considered as statistically significant. Healthy controls: healthy women with normal pregnancy history; TPO + controls: anti-TPO positive women with normal pregnancy history; TPO + RPL: anti-TPO positive women with RPL; BMI: Body Mass Index.

### Th17 and Tregs frequency evaluation

**Fig. 1:**
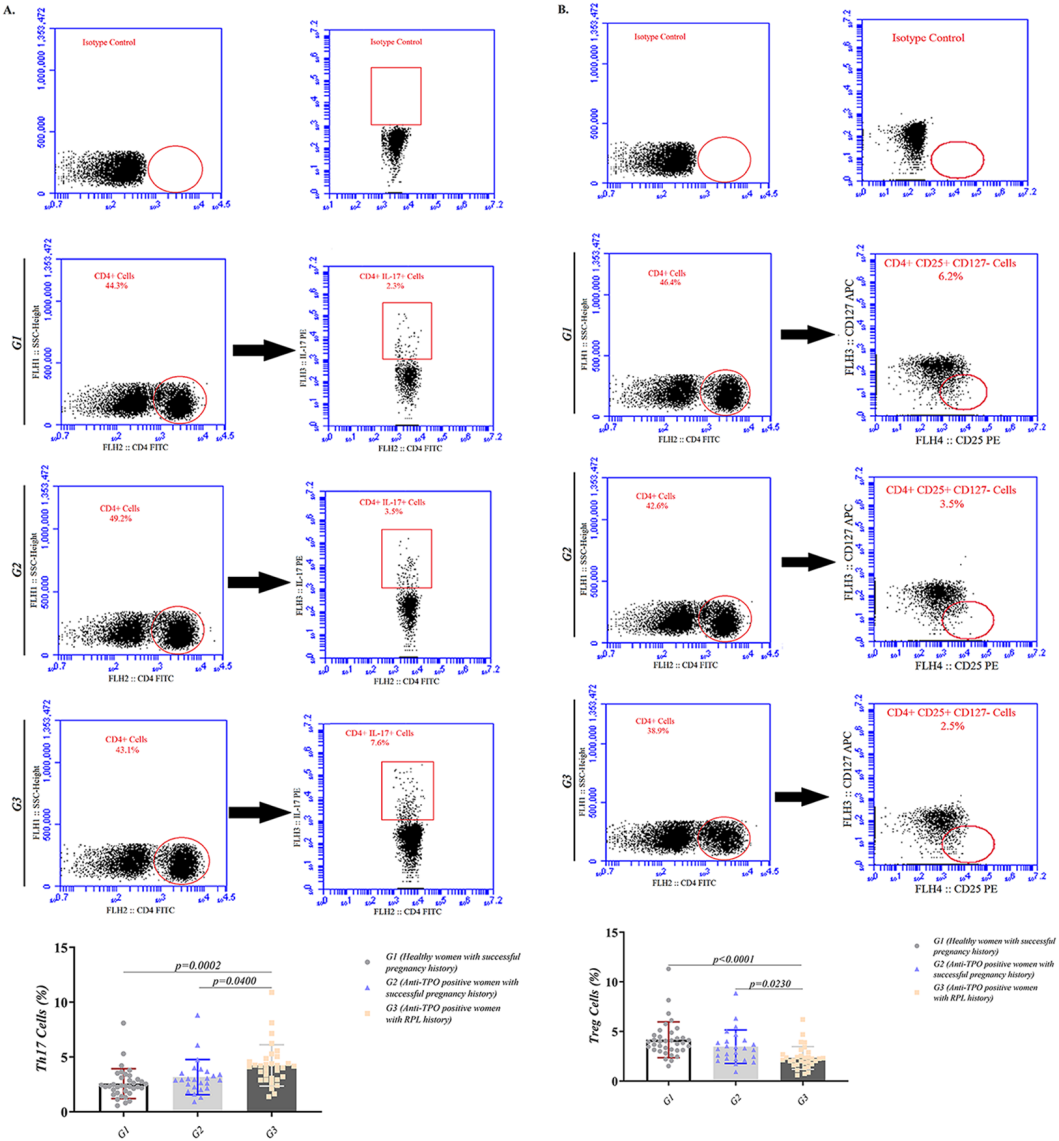
The frequency of Th17 and Tregs measured by flow cytometry technique in healthy controls (n = 36), TPO + controls (n = 25) and TPO + RPL (n = 32) groups. **(A)** Representative dot plots demonstrating the used analyzing method for enumeration of Th17. **(B)** Tregs frequency in studied population. Data are presented as mean ± SD and analysis was done using ANOVA flowed by Turkey’s post-hoc test. The statistical significance was considered as p < 0.05

As it is presented in Fig. 1A and Table S1, Th17% was significantly higher in TPO + RPL compared to healthy controls and TPO + controls (4.236%±1.888% versus 2.572%±1.362%, p = 0.0002 and 3.164%±1.604%, p = 0.04, respectively). In contrast, the percent of Tregs cells was significantly lower in TPO + RPL compared to healthy controls and TPO + controls (2.334%±1.152% versus 4.164%±1.804%, p < 0.0001 and 3.463%±1.680%, p = 0.0230, respectively) (Fig. 1B and Table S1).

### Expression level of immune system related genes

**Fig. 2:**
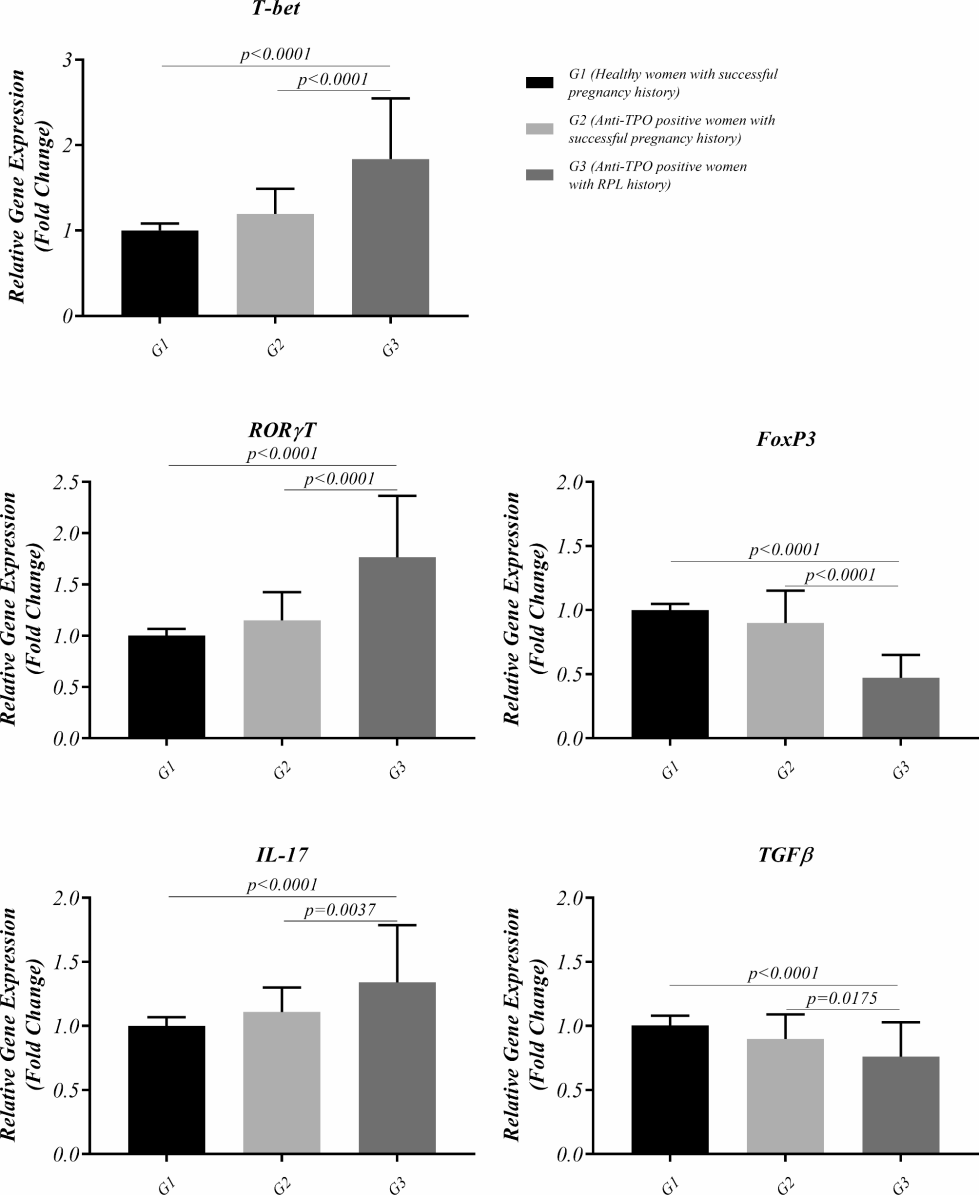
mRNA expression level of RORγT, Foxp3, IL-17 and TGFβ measured by Real-time PCR in healthy controls (n = 36), TPO + controls (n = 25) and TPO + RPL (n = 32) groups. Data are presented as mean ± SD and analysis was done using ANOVA flowed by Turkey’s post-hoc test. The statistical significance was considered as p < 0.05

Quantitative real-time PCR was applied to evaluate the gene expression level of T-bet, RORγT, Foxp3, IL-17 and TGFβ as immune system related genes. As it is illustrated in Fig. 2 and Table S1, RORγT expression was considerably higher in TPO + RPL compared to healthy controls and TPO + controls (1.766 ± 0.6 fold versus 1.000 ± 0.066 fold, p < 0.0001 and 1.152 ± 0.275 fold, p < 0.0001, respectively). Similar results were also observed regarding the IL-17 in mentioned groups (1.340 ± 0.447 fold versus 1.000 ± 0.068 fold, p < 0.0001 and 1.110 ± 0.192 fold, p = 0.0037, respectively). In contrast, a significantly lower Foxp3 expression was observed in TPO + RPL compared to healthy controls and TPO + controls (0.472 ± 0.178 fold versus 1.000 ± 0.049 fold, p < 0.0001 and 0.899 ± 0.253 fold, p < 0.0001, respectively). Similar results were also observed in TGFβ between mentioned groups (0.762 ± 0.267 fold versus 1.004 ± 0.077 fold, p < 0.0001 and 0.899 ± 0.191 fold, p = 0.0175, respectively). Additionally, T-bet expression as a Th1-associated transcription factor, which is affected by Tregs was evaluated in studied population. In this regard, T-bet expression was considerably higher in TPO + RPL compared to healthy controls and TPO + controls (1.836 ± 0.71 fold versus 1.000 ± 0.082 fold, p < 0.0001 and 1.193 ± 0.295 fold, p < 0.0001, respectively).

### Evaluation of cytokines secretion in media supernatant

To evaluate the secretion level of IL-17 and TGFβ in media supernatant of cultured PBMCs, ELISA technique was used. As shown in figure S1 and Table S1, a significantly higher IL-17 secretion was observed in TPO + RPL compared to healthy controls and TPO + controls (146.2 ± 48.44 pg/ml versus 94.50 ± 30.13 pg/ml, p < 0.0001 and 114.1 ± 34.31 pg/ml, p = 0.0072, respectively). However, TGFβ was meaningfully lower in TPO + RPL compared to mentioned groups (308.6 ± 159.3 pg/ml versus 501.9 ± 208.4 pg/ml, p = 0.0002 and 437.8 ± 193.6 pg/ml, p = 0.0359, respectively).

### Correlation analysis between anti-TPO level and Treg or Th17 frequency

Figure S2 shows the correlation analysis between anti-TPO values and Treg or Th17 frequency in anti-TPO positive groups. As it is presented, significant positive and negative correlations were observed between anti-TPO positive women with RPL history and Th17 (r = 0.4784, p = 0.0056) and Treg (r=-0.4229, p = 0.0159), respectively. No significant correlation was observed between anti-TPO positive women with successful pregnancy history and Th17 or Treg frequency.

## Discussion

RPL is a common complication during pregnancy, and different issues such as parenteral chromosomal disorders, anti-phospholipid syndrome, anatomical and environmental factors, thrombophilia and endocrine disorders have been reported to be associated with RPL [18]. In a recent meta-analysis conducted by Xie et al. [7], a significant association was observed between the presence of anti-TPO and increased RPL prevalence.

It has been demonstrated that in situations such as pregnancy, fetal antigens interact with the maternal immune system and Tregs cells dominated the Th17 cell to ensure the fetus survival. In contrast, during autoimmunity Th17 responses are profuse, leading to immune system inflammatory shift and attack to the fetus which has been observed in RPL [19, 20]. Basimi et al. [15] have indicated a link between the presence of anti-TPO and increased Th17/Tregs ratio in RPL patients. Lee et al. [17] in their study on 42 RPL patients and 24 normal controls also reported a higher Th17 and lower Tregs cells frequency in RPL patients.

In the present study three groups of patients including healthy controls, TPO + controls and TPO + RPL were evaluated regarding the immune system components such as Th17 and Tregs cells, cytokines and immune system related genes expression and secretion. Similar to previous studies, our data indicated a significant increase in Th17 and decrease in Tregs cells in TPO + RPL compared to healthy controls and TPO + controls groups.

Our findings indicated a significant increased expression (or secretion) of RORγT and IL-17 in TPO + RPL compared to healthy controls and TPO + controls. Moreover, a decreased expression of FoxP3 was also observed in mentioned group compared to the other groups. These data indicated a higher inflammatory condition in anti-TPO positive women with RPL history. Saifi et al. [21] in a study on 20 spontaneous abortion patients and 20 normal controls also reported a lower FoxP3 expression in patients with spontaneous abortion compared with controls. Previously, the continuous increase of IL-17 during pregnancy has been reported [22].

In the present study our data showed a significant decreased expression and secretion of TGFβ in anti-TPO positive women with RPL history compared to the other groups. There are limited data regarding the level of this cytokine in thyroid autoimmune diseases. As previously mentioned above, saifi et al. [21] also reported a lower TGFβ in RPL patients compared to controls.

## Conclusion

Based on our findings, it can be hypothesized that thyroid autoimmunity may accelerate the inflammatory status and worsen the pregnancy outcomes. We also demonstrated increased level of Th17 frequency, increased RORγT and IL-17 expression and secretion in TPO + controls and TPO + RPL. In contrast, Tregs frequency and FoxP3 and TGFβ were significantly lower in TPO + groups with a higher severity in TPO + RPL.

### Limitations

The low number of participants, especially in TPO + controls group was the main limitation of this study. Additionally, we lack the data regarding T3, T4 and anti-TG in study population.

### Electronic supplementary material

Below is the link to the electronic supplementary material.

Supplementary Material 1. **Table S1.**

Supplementary Material 2. **Figure S1.** Concentrations of TGFβ and IL-17 measured by ELISA method in healthy controls (n = 36), TPO + controls (n = 25) and TPO + RPL (n = 32) groups. Data are presented as mean ± SD and analysis was done using ANOVA flowed by Turkey’s post-hoc test.

Supplementary Material 3. **Figure S2.** Correlation analysis between anti-TPO values and Th17 or Treg frequencies in anti-TPO positive women with successful pregnancy history or RPL. r, pearson correlation coefficient. The statistical significance was considered as p < 0.05.

## Data Availability

The data that support the findings of this study are available on request from the corresponding author. The data are not publicly available due to privacy or ethical restrictions.

## Abbreviations

*ELISA*: Enzyme-linked immunosorbent assay
*FITC*: fluorescein isothiocyanate
*PBMCs*: Peripheral blood mononuclear cells
*RORC*: Retinoid orphan receptor C
*RPL*: Recurrent pregnancy loss
*RT-PCR*: Real-time polymerase chain reaction
*TG*: Thyroglobulin
*TGF-β*: transforming growth factor beta
*TPO*: Thyroid peroxidase
*TSH*: Thyroid stimulating hormone
