# Supplementary material for: The association of Treg and Th17 cells development factors and anti-TPO autoantibodies in patients with recurrent pregnancy loss
Source: BMC Res Notes. 2023 Oct 31;16:302. doi: 10.1186/s13104-023-06579-6 (PMC10619307; doi:10.1186/s13104-023-06579-6)
Supplement: Supplementary file 1 — Supplementary Material 1. Table S1. [file 13104_2023_6579_MOESM1_ESM.docx]

| **Table S1.** Expression level and cell supernatant concentration of immune system related genes and the frequency of Th17 and Treg cells | | | | | | |
| --- | --- | --- | --- | --- | --- | --- |
| ***P* value** | | | **G3 (n=32)**  **mean±SD** | **G2 (n=25)**  **mean±SD** | **G1 (n=36)**  **mean±SD** | **Parameter** |
| **G2 vs G3** | **G1 vs G3** | **G1 vs G2** |  |  |  |  |
| **Gene Expression Level** | | | | | | |
| <0.0001 | <0.0001 | NS | 1.836±0.71 | 1.193±0.295 | 1.000± 0.082 | ***T-bet*** |
| <0.0001 | <0.0001 | NS | 0.472±0.178 | 0.899±0.253 | 1.000± 0.049 | ***FoxP3*** |
| <0.0001 | <0.0001 | NS | 1.766±0.6 | 1.152±0.275 | 1.000±0.066 | ***RORγT*** |
| 0.0037 | <0.0001 | NS | 1.340±0.447 | 1.110±0.192 | 1.000±0.068 | ***IL-17*** |
| 0.0175 | <0.0001 | NS | 0.762±0.267 | 0.899±0.191 | 1.004±0.077 | ***TGFβ*** |
| **Cell Supernatant ELISA** | | | | | | |
| 0.0072 | <0.0001 | NS | 146.2±48.44 | 114.1±34.31 | 94.50±30.13 | **IL-17 (pg/ml)** |
| 0.0359 | 0.0002 | NS | 308.6±159.3 | 437.8± 93.6 | 501.9±208.4 | **TGFβ (pg/ml)** |
| **Flow cytometry** | | | | | | |
| 0.0400 | 0.0002 | NS | 4.236±1.888 | 3.164±1.604 | 2.572±1.362 | **Th 17 (%)** |
| 0.0230 | <0.0001 | NS | 2.334±1.152 | 3.463±1.680 | 4.164±1.804 | **T_reg_ (%)** |
| Data are presented as mean ± standard division (SD). p<0.05 was considered as statistically significant. G1: healthy women with normal pregnancy history; G2: anti-TPO positive women with normal pregnancy history; G3: anti-TPO positive women with RPL; T-bet: T-box transcription factor TBX21; FoxP3: Forkhead box P3; history; IL-17: Interlukin-17; RORγT: RAR-related orphan receptor gamma; TGFβ: Transforming growth factor beta; Treg: Regulatory T cell. | | | | | | |
