# Supplementary figures and images for: The association of Treg and Th17 cells development factors and anti-TPO autoantibodies in patients with recurrent pregnancy loss
Source: BMC Res Notes. 2023 Oct 31;16:302. doi: 10.1186/s13104-023-06579-6 (PMC10619307; doi:10.1186/s13104-023-06579-6)

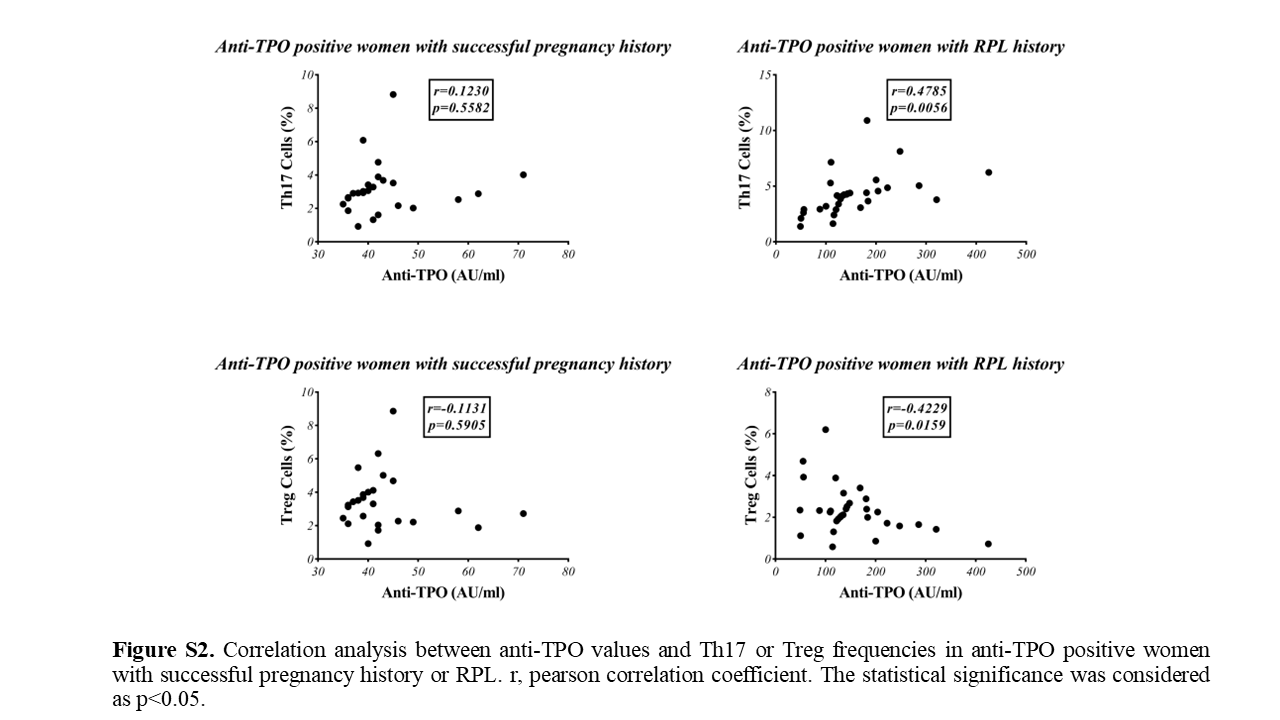

Supplement: Supplementary file 2 — Supplementary Material 2. Figure S1. Concentrations of TGFβ and IL-17 measured by ELISA method in healthy controls (n = 36), TPO + controls (n = 25) and TPO + RPL (n = 32) groups. Data are presented as mean ± SD and analysis was done using ANOVA flowed by Turkey’s post-hoc test. [file 13104_2023_6579_MOESM2_ESM.tif]

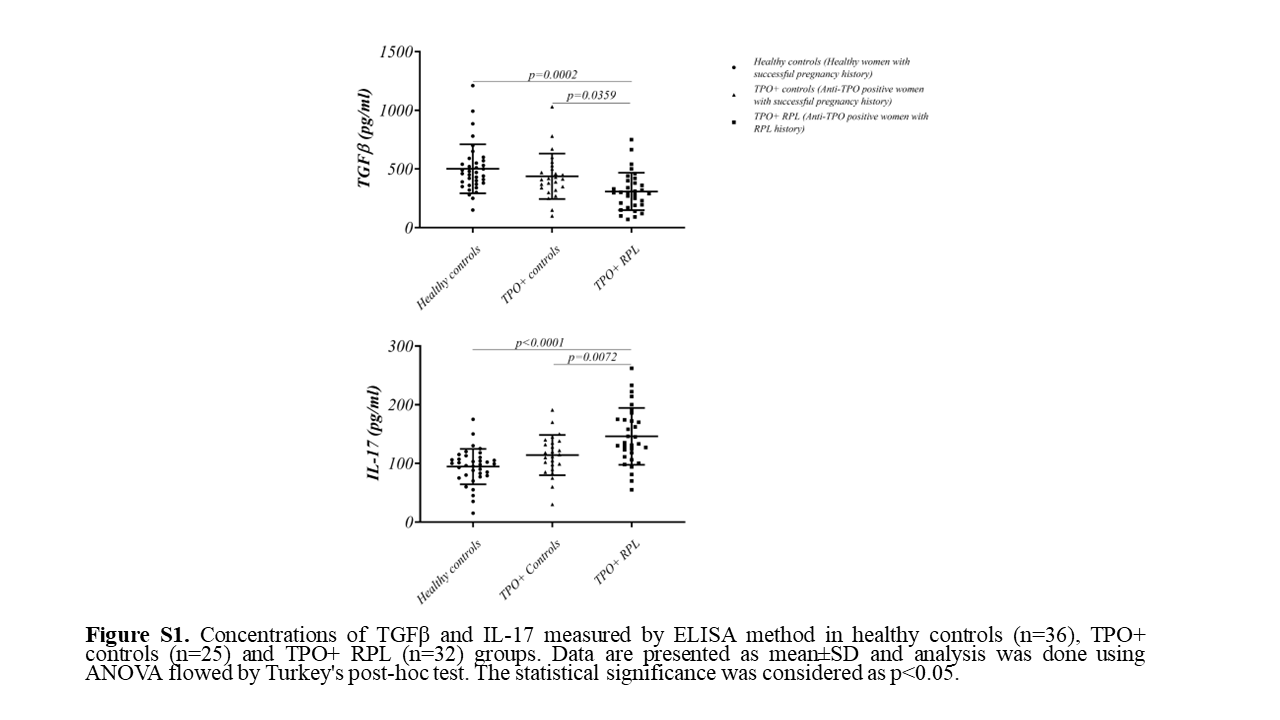

Supplement: Supplementary file 3 — Supplementary Material 3. Figure S2. Correlation analysis between anti-TPO values and Th17 or Treg frequencies in anti-TPO positive women with successful pregnancy history or RPL. r, pearson correlation coefficient. The statistical significance was considered as p < 0.05. [file 13104_2023_6579_MOESM3_ESM.tif]
